# Characteristics, treatments and outcomes in patients with severe burn wounds; a 10 year cohort study on acute and reconstructive treatment

**DOI:** 10.1371/journal.pone.0313287

**Published:** 2024-11-22

**Authors:** Lucindi Smit, Anouk Pijpe, Cindy Nguyen, Tara Hartsuiker, Matthea Stoop, Anouk van Heel, Eelke Bosma, Cornelis H. van der Vlies, Paul P. M. van Zuijlen, Margriet E. van Baar, Esther Middelkoop, Dutch Burn Repository group

**Affiliations:** 1 Department of Plastic, Reconstructive and Hand Surgery, Amsterdam UMC location Vrije Universiteit Amsterdam, Amsterdam, The Netherlands; 2 Amsterdam Movement Sciences (AMS), Tissue Function and Regeneration, Amsterdam UMC, Amsterdam, The Netherlands; 3 Alliance of Dutch Burn Care (ADBC) and Burn Centre, Red Cross Hospital, Beverwijk, The Netherlands; 4 Alliance of Dutch Burn Care (ADBC) and Burn Centre, Martini Hospital, Groningen, The Netherlands; 5 Alliance of Dutch Burn Care (ADBC) and Burn Centre, Maasstad Hospital, Rotterdam, The Netherlands; 6 Erasmus MC, Trauma Research Unit, Department of Surgery, University Medical Center Rotterdam, Rotterdam, The Netherlands; 7 Department of Plastic and Reconstructive Surgery, Red Cross Hospital, Beverwijk, The Netherlands; 8 Pediatric Surgical Centre, Emma Children’s Hospital, Amsterdam UMC location University of Amsterdam, Amsterdam, The Netherlands; 9 Department of Public Health, Erasmus MC, University Medical Centre Rotterdam, Rotterdam, The Netherlands; 10 Research Group Healthy Ageing, Allied Health Care and Nursing, Hanze University of Applied Sciences, Groningen, The Netherlands; 11 Center for Human Movement Sciences, University of Groningen, University Medical Center Groningen, Groningen, The Netherlands; Uniformed Services University of the Health Sciences, UNITED STATES OF AMERICA

## Abstract

Reports on treatment characteristics and long term outcomes for severe burns are scarce, while the need to compare outcomes of novel treatment modalities to standard of care is increasing. Our national database on burn treatment enabled analysis of patient as well as treatment characteristics during acute treatment and following reconstructive procedures. Furthermore, outcome data of longitudinal scar assessments were analysed from a single burn centre database. Acute and reconstructive data were analysed for patients admitted to the three Dutch burn centres with total body surface area burned of ≥ 20% TBSA. Long term outcome was analysed from a single centre scar database, both for a period of 2009–2019. Treatment characteristics from 396 surviving acute burn patients were analysed. Surgical treatment was required in 89.6% of these patients and 110 patients (27.8%) needed reconstructive surgery in the years after the burn incident, with a mean of 4.4 reconstructive procedures per patient. Main indications were contractures (70.5%) and arms (45.0%) and head and neck region (41.2%) were most frequently affected. Techniques used for reconstructive corrections were predominantly excision, release and flaps (54.7%), followed by skin transplants (32.4%). Scar quality was significantly worse in patients with more severe burns compared to those with TBSA < 20% during prolonged times. These data provide insight into health care utilization, treatment characteristics and outcomes in severely burned patients. These real-world data can guide future development of improved treatment strategies for at risk patients as well as anatomical locations.

## Introduction

Despite great advances in burn care [1, 2] treating severe burns and burn defects remains challenging with many different treatment options and varying outcomes. Burns often result in significant scarring, cosmetic disfigurement and functional disability. Many severely burned patients need multiple operations to prevent and treat complications in both the acute setting and long term [3–5].

When the total body surface area (TBSA) burned increases and the dermal layer and its extracellular matrix are severely injured, as is the case in deep dermal and full-thickness burns, the skin cannot regenerate. As these defects heal, they often form large scars and in some cases these will contract causing significant functional problems [3, 6–8].

The formation of scar tissue is a significant and complex problem. Since several decades, focus of burn treatment is on limiting scar formation. The current gold standard for the treatment of large deep dermal and full-thickness burns is the application of a split-thickness autograft: a thin layer of skin containing the epidermis and a small part of the dermis, often expanded by mesh or MEEK techniques [9, 10]. However, techniques such as these also have disadvantages. One aspect is that for harvesting split skin a donor site is needed which is not always readily available in patients with extensive burns. The donor site itself is an additional large wound with accompanying risk of infection, wound healing problems and scar formation and imposes substantial discomfort for the patient [11–13]. Furthermore, currently no treatment provides full regeneration of these deep burn wounds. Therefore, novel treatment strategies are continuously being tested in order to improve the regeneration capacity for these severe defects. Some of these strategies are: dermal substitutes, used in combination with split-thickness autografts, cultured epidermal or full skin constructs or techniques such as Recell®, where epidermal cells are harvested during surgery and sprayed on the wound area [14–20].

When evaluating the effectiveness of these novel treatments data are needed to compare outcomes of novel treatment modalities to current standard of care treatment. However, when searching for comprehensive and current baseline data we found that there is minimal baseline data available on the standard treatment, especially for the category of patients with large area burns (≥ 20% TBSA [21, 22]) and their outcomes. Next to health care outcomes such as survival rates, length of stay and complication rates [22–25], long term outcomes in terms of scar quality are important. Several assessment tools are available to measure scar quality [26, 27]. Studies that have sought to describe trends in the treatment of burns provide limited data on a wide variety of procedures, scar quality and focus on smaller TBSA ranges [28–30]. One retrospective study conducted in 2014 evaluated the reconstructive needs of 1768 burn patients over a 10-year period [31]. In this study, 13% of patients required reconstructive surgery during the 10-year follow-up period. Mean percentage TBSA burned in this subgroup that required reconstructions was 20.6 ± 18.7 and locations that were most frequently reconstructed were hands, head and neck. Studies concerning scar quality in burn patients have shown that a high percentage TBSA burned and more operations [32–34] both contribute to a reduction in scar quality, however for severely burned patients there is very little baseline data available on the treatment pathway and the resources used and how these contribute to scar quality over time.

To innovate the field of burn care an overview is needed of the effectiveness and outcomes of the current treatment strategies implemented in the acute setting but also providing data on long term outcomes. This baseline data will not only provide insight into how effective our current treatment strategies are, but it will also allow us to accurately compare outcomes of novel treatment strategies with existing ones in future studies. Furthermore, such data could provide more insight into the areas where improvement is most needed.

The purpose of this study is threefold: 1) to catalogue the use of care in patients with extensive severe burns admitted for care to one of the three Dutch burn centres in an extended time period; 2) to describe in more detail the characteristics and health care utilization in the reconstructive phase of burn treatment within a minimal time post burn of 3 years; 3) to evaluate outcome of wound healing in terms of observer- and patient-reported scar quality over time.

## Materials and methods

### Study design and data collection

For this multicentre cohort study, data was extracted that had been collected between 2009 and 2022 from the Dutch Burn Repository-R3 (DBR-R3). Data were accessed on May 20, 2022. The DBR-R3 database contains data on all patients admitted to one of the three burns centres in the Netherlands; the Red Cross Hospital Beverwijk, Martini Hospital Groningen and Maasstad Hospital Rotterdam. Researchers had no access to patient-identifying information. Data concerning scar quality was obtained from the scar registry from the Red Cross Hospital. These data were collected on April 9, 2024. The two other burns centres in the Netherlands did not maintain such records in this period.

This study was conducted according to the guidelines of the International Conference on Harmonization—Good Clinical Practice. The Medical Ethics Review Committee of the Amsterdam University Medical Centers ruled that the Medical Research Involving Human Subjects Act (WMO) was not applicable to the current study (2021.0446 and 2024.0112). The study was approved by the institutional review board of the three hospitals. Patient consent was obtained through an opt-out procedure to register their data for scientific research.

### Patient selection

Patients were included if they were admitted between 2009 and 2019 to one of the three Dutch Burn Centres with burn wounds exceeding 20% of their body surface area, with a clinical admission time of more than 2 hours. Using data on reconstructive surgery up to 2022, all patients had a follow-up of at least 3 years. Patients were excluded for treatment analysis if death had occurred within four days after the burn incident or during the acute treatment phase.

From the scar registration database, data from patients with a burn incident in the period 2009 to 2019 was extracted using the same inclusion and exclusion criteria, however, without the selection of 20% TBSA. In this way, a comparison could be made for scar outcome for patients with burns < 20% TBSA and above.

### Data extraction

Demographic data including sex, year of burn incidence and age at the time of burn incidence were extracted, in addition to data on percentage TBSA burned, timing and types of procedures carried out, length of hospital stay (LOS) and the occurrence rate of wound infections or other complications and co-morbidities.

When a patient had undergone multiple reconstructive surgeries, data was collected on the number of admissions and details of the surgical revision (number of surgeries, details on procedures performed, techniques, indications, locations and timing). Scar quality outcomes were measured with the Patient and Observer Scar Assessment Scale (POSAS) [35]. This numeric scale consists of two questionnaires: one completed by the patient and other by the observer (trained specialist, nurse, researcher or therapist). The responses to these questionnaires were collated and compared across 4 time periods; 0–4.5 months, between 4.5 and 9 months, 9–15 months and 15–21 months. Scores above 21 months were not analysed as they were too few in numbers.

### Statistical analysis

Analyses were performed using Statistical Products and Service Solutions (SPSS) 25.0 for Windows (SPSS Inc., Chicago, IL). POSAS score outcomes were analysed separately for two subgroups: mild/intermediate burns (% TBSA 0–19.9) and severe burns (≥20% TBSA). Continuous variables were presented as mean ± standard deviation and median (range). Comparisons of continuous variables within groups (survivors vs. non survivors in the acute phase, reconstructive vs. no reconstructive surgery and scar quality in patients with mild/intermediate and severe burns) were analysed with a Mann-Whitney U-test. To further analyze the time to the first reconstructive surgery among survivors (n = 396), a Kaplan-Meier one-minus-survival analyses was also performed. The time to event variable was the interval between the date/year of injury to the date of the event or the last follow-up time (31-12-2022), whichever occurred first.

## Results

### Inclusions

**Fig 1:**
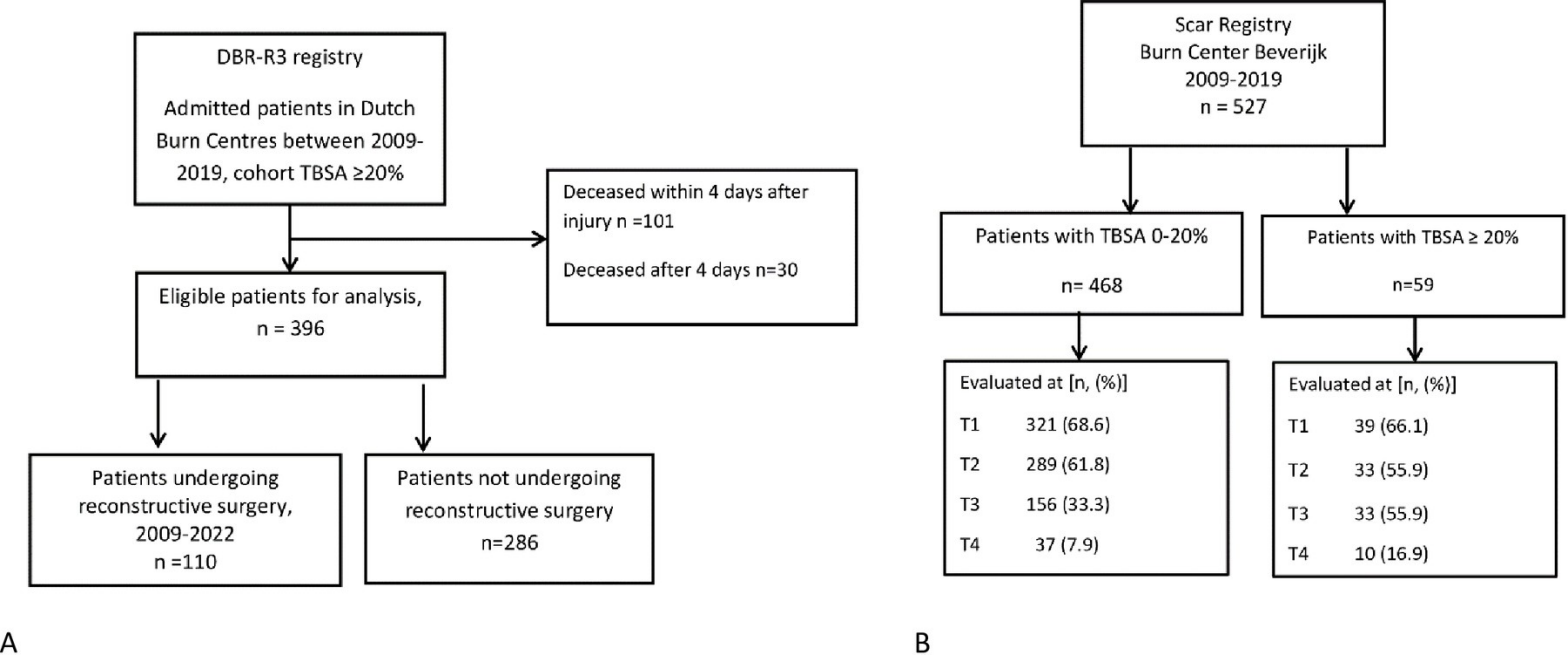
A. Flowchart patient inclusion DBR-R3 Registry, B. Flowchart patient inclusion Scar Registry. Number of patients analysed form DBR-R3 Registry (A) and Scar Registry (B). Timepoints of analysis: T1 0–4.5 month, T2 4.5–9 months, T3 9–15 months, T4 15–21 months.

In total, 527 patients had been admitted to one of the three Dutch burn centres with burns ≥ 20% TBSA during the 11-year period (Fig 1A). A total of 396 (75.1%) surviving burn patients were identified. Of the 131 deceased patients, 101 died within 4 days after the injury. Of the remaining 30 patients, in 17 cases further treatment was refrained. Patient characteristics are described in Table 1.

**Table 1:** Baseline characteristics of hospitalized burn patients.

Number patients (%)	Total	Survivors included for analysis	*Non-survivors*	*p-value* ^*a*^
	*n = 527 (100)*	*n = 396 (75*.*1)*	*n = 131 (24*.*9)*	
Age (years)				
Mean age ± SD	43.8 ± 21.8	38.1 ± 19.7	61.0 ±18.6	0.000
Median age (range)	44 (1–98)	38 (1–94)	59.0 (12–98)	
Sex (*n*, %)				
Male (%)	368 (69.8)	286 (72.2)	82 (62.6)	0.038*
Female (%)	159 (30.2)	110 (27.8)	49 (37.4)	
% TBSA burned				
Mean ± SD	40.3 ± 20.2	34.6 ± 15.1	57.7 ± 23.5	0.000
Median (range)	34 (20–100)	30.0 (20–95)	56.0 (20–100)	
LOS (days)^b^				
Mean ± SD	44.2 ± 43.8	55.9 ± 43.1	8.53 ±20.8	0.000
Median (range)	34.0 (0–383)	45.0 (0–383)	1.00 (0–121)	
Baux score				
Mean ± SD	84.1 ± 31.3	72.6 ± 24.9	119 ± 21.6	0.000
Median (range)	84 (21–178)	73.0 (21–148)	119 (40–178)	

P-values calculated with a Mann-Whitney U test

* ANOVA test

^a^ P-difference between the subgroups survivors and non-survivors

^b^ Total LOS, length of hospital stay during first admission, readmission(s) and transfer between burn centres if applicable

From the scar registry data were collected only in Red Cross Hospital between 2009 and 2019, 918 measurements of scar data from 527 patients were collected, 468 had TBSA below 20% and 59 had TBSA ≥ 20% (Fig 1B).

### Study population and treatment characteristics of patients admitted for acute burn treatment

Characteristics of the surviving and non-surviving patients are reported in Table 1. Non-survivors, including comfort care patients, were older in age, had a higher TBSA percentage burned and a shorter length of hospital stay (*p* = 0.000). Males accounted for the majority of hospitalized burn patients with a total number of 368 (69.8%). Baux scores [36] were calculated as the sum of age and TBSA of the patient and was significantly different between survivors and non-survivors.

The final study sample of surviving burn patients consisted of 396 patients. Table 2 provides details on patient, burn and treatment characteristics. Among the final sample, patients had a mean age of 38.1 ± 19.7 years at the time of burn injury with a mean percentage TBSA burned of 34.6 ± 15.1 (range 20.0–95.0). Most patients presented with a TBSA burned of 20–30%, and burn wound infection occurred in 8.1% of the total sample. Mean length of hospital stay including readmissions during the acute phase was 55.9 ± 43.1 days.

**Table 2:** Burn and treatment characteristics of surviving burn patients.

	Included patients
	*n* = 396
% TBSA burned (n, %)	
20–30	204 (51.5)
31–40	91 (23.0)
41–50	39 (9.8)
50 +	58 (14.6)
Surgical treatment (*n*, %)	355 (89.6)
Techniques (*n*, %)	
Meshed STSG	297 (75.0)
Unmeshed STSG	11 (2.8)
Meek STSG	155 (39.1)
FTG	13 (3.3)
Allograft skin	99 (25.0)
Other transplants ^a^	12 (3.2)
Sandwich graft ^b^	4 (1.0)
Dermal substitutes	13 (3.3)
Topical Negative Pressure	11 (2.8)
Primary closure	7 (1.8)
Wound infection (*n*, %)	32 (8.1)

Percentages presented as percentage within subgroup

^a^ Other transplants included the use of autologous cultured proliferating epidermal cells, transpositions, perforator flaps and application of platelet-rich plasma.

^b^ *Sandwich graft*, allograft overlay to temporarily cover burn wounds treated with autografts.

Surgical treatment of the burn wound was indicated in 89.6% of cases (Table 2). The most frequently applied techniques were the use of meshed split-thickness skin grafts (75%), followed by meek split-thickness skin grafts (39.1%) and allograft skin (25.0%).

### Reconstructive surgery

In 110 (27.8%) of the surviving patients, surgical revision of the burn scar was performed during the period 2009–2022. Table 3 shows the differences in characteristics between patients who underwent reconstructive surgery after the acute phase and who did not. Patients who underwent reconstructive surgery, had a higher % TBSA burned, higher Baux scores and a longer LOS during the acute phase (*p* < 0.025). Patients showed no significant difference in mean age at the time of burn injury (*p* = 0.972). Details on number of surgeries, which could include several procedures performed during the same operative session, and the most treated location per admission are reported in Tables 4 and 5. A total of 475 surgical procedures were performed in 282 hospital admissions. A large number of patients underwent one reconstructive admission (40.9%).

**Table 3:** Characteristics of patients with and without reconstructive surgery.

	All patients	Reconstructive surgery	No reconstructive surgery	*p-value*
Total (*n*,*%)*	396 (100)	110 (27.8)	286 (72.2)	
Sex (*n*,*%)*				
Male	286 (72.2)	73 (66.4)	213 (74.5)	0.107*
Female	110 (27.8)	37 (33.6)	73 (25.5)	
Mean age at burn injury ± SD	38.1 ± 19.7	38.1 ± 18.2	38.0 ± 20.3	0.972
Mean % TBSA ± SD	34.6 ± 15.1	39.0 ± 14.6	32.9 ± 15.0	<0.001
Mean LOS ± SD	55.9 ± 43.1	82.2 ± 52.4	45.8 ± 34.0	<0.001
Baux score	72.6 ± 24.9	77.1 ± 24.9	70.9 ± 25.5	0.025

P-values calculated with a Mann-Whitney U test.

*LOS*, length of hospital stay including readmissions in the acute phase.

*ANOVA

**Table 4:** Characteristics of reconstructive admissions.

	All patients
Total (*n*,*%)*	110 (100)
Admissions for reconstructive surgery	282
Mean ± SD	2.6 ± 2.0
Median (*range*)	2.0 (1–10)
Reconstructive procedures	475
Mean ± SD	4.4 ± 4.3
Median (*range*)	3 (1–29)
Number of reconstructive admissions	
1	45 (40.9)
2	24 (21.8)
3	17 (15.5)
4	9 (8.2)
>4	15 (13.6)
Time to first reconstructive surgery (days)	
Mean ± SD	461.9 ± 376.5
Median (*range*)	384 (80–2106)

**Table 5:** Details of performed surgical reconstructive procedures: Locations, indications and techniques.

Number of procedures performed	All patients (110)
	*n* = 475
**Location** (*n*,*%)*	***515 (100)***
*Head and neck*, *total*	**212 (41.2)**
Ears	13 (2.5)
Upper eyelids	7 (1.4)
Lower eyelids	18 (3.5)
Nose	7 (1.4)
Mouth	42 (8.2)
Face (NOS ^**a60**^^**a**^)	28 (5.4)
Neck	97 (18.8)
*Arms*, *total*	**232 (45.0)**
*Trunk*	**33 (6.4)**
*Legs*, *total*	**38 (7.4)**
**Indication** (*n*,*%*)	***518 (100)***
*Contractures*, *total*	**365 (70.5)**
Webspace	30 (5.8)
Ectropion	6 (1.2)
Contracture (NOS ^*a*^)	329 (63.5)
*Scar problems*	**120 (23.2)**
Persistent defect	26 (5.0)
Hypertrophic scar	89 (17.2)
Scar Relief	5 (1.0)
*Cosmetic*	**33 (6.4)**
Pigment/colour	4 (0.8)
Contour nose/ear	11 (2.1)
Other ^b^	18 (3.5)
**Technique** (*n*,*%*)	***590 (100)***
*Excision*, *release and flaps*	**323 (54.7)**
*Skin grafting*, *transplant*	**191 (32.4)**
Split skin graft	76 (12.9)
Full thickness graft	113 (19.2)
Dermal substitute + graft	2 (0.3)
*Other techniques*	**76 (12.9)**
Corticosteroid injection	30 (5.1)
Lipofilling	9 (1.5)
Microreleasing	20 (3.3)
Arthrodesis	3 (0.5)
Arthrolysis/Tenolysis	7 (1.2)
Others ^c^	7 (1.2)

*Note*. Numbers of main categories are outlined in bold numbers.

^*a*^ *NOS*, not otherwise specified.

^b^ Others included: alopecia, correcting anatomical symmetry and microstomia.

^c^ Others included: microneedling, lipectomy

### Timing and number of reconstructive surgeries

**Fig 2:**
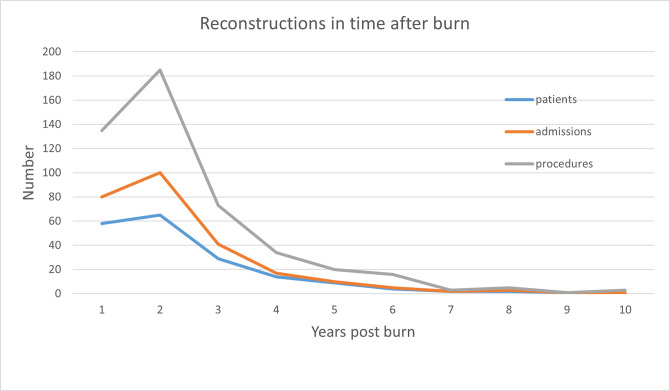
Reconstructions in time after burn. Number of reconstructions in the years after burn, indicated are numbers of patients undergoing reconstruction(s) in the indicated year (blue), number of admissions (orange) and number of procedures (grey).

Patients had a mean number of 4.4 ± 4.3 reconstructive procedures (range 1–29). The first reconstructive surgery was performed most frequently within one year post burn injury (58/110 patients) (Fig 2, S1 Fig). Mean timing until first reconstruction was 461.9 ± 376.5 days (1.3 ± 1.0 years) post burn (Table 4). Most reconstructive procedures were performed within two years post burn: 28.4% within one year and an additional 38.9% between one and two years (Fig 2).

### Location, indication and technique of reconstruction

In Table 5 an overview of types of procedures in the performed surgeries is shown. Reconstructive procedures most often involved the arms (45.0%), followed by the head and neck area (41.2%).

Scar contractures (70.5%) were the most common indication for reconstructive surgery. Other indications included hypertrophic scarring (17.2%), a persistent defect (5.0%) and cosmetic reasons (6.4%). The most frequently applied techniques were excision, release and flaps (54.7%), followed by the use of skin grafts and transplants (32.4%). Various other techniques (12.9%) were further specified in the dataset, such as corticosteroid injections (5.1%), micro-releasing (3.3%) and arthrolysis/tenolysis (1.2%).

### Scar quality–patient and observer scores

Results of the patient and observer POSAS scores are presented in Table 6 and S1 Table. Data on sex, age and % TBSA burned was analysed for 527 patients. At 4 time points, clinicians/researchers and patients evaluated scar quality: time point 1 (between 0–4.5 months), time point 2 (between 4.5–9 months), timepoint 3 (between 9 and 15 months) and timepoint 4 (15–21 months). The number of patients that could be evaluated gradually decreased in time.

**Table 6:** Characteristics of study population for scar evaluation.

	Total	TBSA 0–20	TBSA ≥20	P-value
	*n = 527*	*n = 468*	*n = 59*	
**Sex, (*n*,*%)***				
Male	324 (61.5)	283 (60.5)	41 (69.5)	0.180
Female	203 (38.5)	185 (39.5)	18 (30.5)	
**Age at burn**				
Mean ± SD	27.5 ± 23.3	26.4 ± 23.3	36.5 ± 21.0	0.001
Median (range)	23.4 (0–82)	20.6 (0–82)	37,9 (0,8–75)	
**% TBSA burned**				
Mean ± SD	9.10 ± 10.6	6.0 ± 4.42	33.6 ± 13.3	0.000
Median (range)	6.0 (0.2–96)	5.0 (0.2–19.5)	33.0 (20–96)	
**Baux scores**				
Mean ± SD	36.0 ±26.7	31.8 ±23.8	69.6 ± 24.7	0.000
Median (range)	32.5 (0.5–124)	28.0 (0.5–90)	69.0 (21–124)	

P-values for differences between groups TBSA <20% and TBSA ≥ 20%, calculated with Mann Whitney or ANOVA for categorical data

Mean time between the initial burn injury and moment of scar evaluation did not significantly differ between patients with burns of 0–20% TBSA or ≥ 20% TBSA. In general, POSAS scores reported by patients were higher compared to observer given scores.

**Fig 3:**
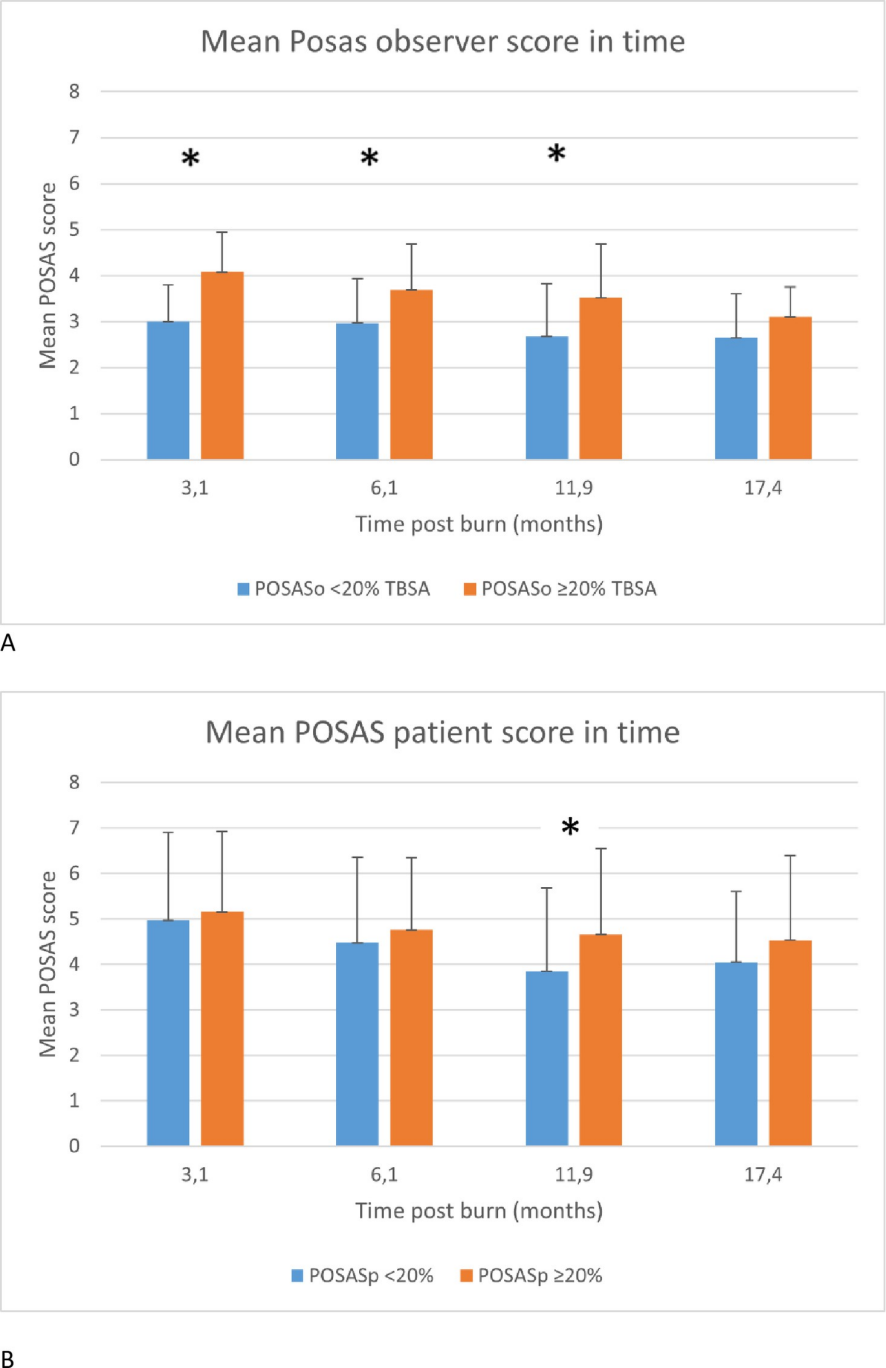
A. Mean POSAS observer score in time, B. Mean POSAS patient score in time. * = statistically significant difference between score in group <20% TBSA versus group ≥20% TBSA.

The mean observer POSAS score was significantly higher (meaning worse scars) in patients with severe burns (*p* ≤ 0.001) for all time points until 18 months, indicating lower scar quality compared to observer POSAS scores of burns under 20% TBSA. A trend in higher mean POSAS scores was also found in patients with severe burns, but significance was only reached at timepoint 3 (9–15 months post burn). In both groups, mean POSAS scores started decreasing after the first measurements. Fig 3A and 3B show a graphical display of the mean POSAS scores in time.

## Discussion

This multicenter cohort study elucidated the healthcare utilization in the acute and reconstructive phase for severely burned patients over a > 10-year time period. The results of this study can be summarized as follows: 1) the mortality rate in the acute phase was 24.9% for patients with a mean Baux score of 119; 2) mean length of stay of surviving patients was 55.9 days; 3) the prevalence of reconstructive surgery after acute burns was 27.8% with patients undergoing a mean number of 4.4 ± 4.3 reconstructive surgeries; 4) scar severity was rated significantly higher in patients with severe burns compared to scores in patients with less severe burns.

Few studies have been published on the health care utilization in severely burned patients, and especially ‘real-world data’ of patients outside of specific trials are lacking. One observational prospective study by Klein et al. [28] collected outcome data in the acute phase of 541 patients with burns exceeding 20% TBSA. Patients were categorized as paediatric patients (0–17 years) or adult patients (>17 years). This study was conducted from May 2003 to February 2010 in 6 academic US burn centres and patients were treated according to pre-established ‘standard operating procedures’ (SOPs) to guarantee uniform and homogenous care. What these SOPs specifically entailed and how conformity to these protocols was assured, was not further specified in the report. Although the study of Klein et al. states that patients were included if burns required surgical treatment, these procedures were not further specified. Mean % TBSA burned in non-survivors was 50.0 ± 18.1 and 8.3 ± 6.0 in adults and children respectively. Our study did not stratify survivors and non-survivors by age as our sample contained only one non-surviving paediatric patient. Our findings that non-survivors were older, more severely injured and had a shorter admission time are in line with the results reported in the study from Klein et al. [28] When calculated for the total population, mortality rate is evidently lower than in our study: 13.6% vs. 24.9% respectively. Possibly, the lower % TBSA burned and shorter study period are responsible for this difference. Another explanation could be that included patients had a higher survival rate due to the criterium of operative treatment being required in the study of Klein et al. Consequently, this criterium excludes patients who died shortly after the burn incident, on which our study does report. In our study, mortality in the group of patients who survived beyond the initial 4 days after the burn was 5.7%. A systematic review by Brusselaers et al. [22] reported mortality rates between 1.4 and 18%, but in this review studies were included with mean TBSA of 11 to 25% overall, and ‘severe burns’ were defined as ‘an acute burn injury in need of specialized care during hospital admission’.

Similar findings concerning location and indication of reconstructive surgeries performed have been reported in an earlier study performed by Hop et al. [31] This retrospective study included all patients admitted to one of the three Dutch burn centres from January 1998 to December 2001 and assessed the need for revision surgery in a 10-year follow-up period. Hop et al. also observed more reconstructions of the arms and head/neck area and the most frequently reported indication was scar contracture. This was also found in a detailed analysis of Schouten et al. [8] These authors explained the lower incidence of contractures in lower extremities by mobilization of patients in the upright position during rehabilitation, leading to improvements in range of motion of hip and knee joints over time. The prevalence of reconstructive surgery shown in our study was higher than the reported prevalence in Hop et al: 27.8% vs. 13.0%. A possible explanation could be the difference in defined cohorts of patients and the dissimilarities in patient characteristics. The study of Hop et al [31] described a study sample of 229 patients with mild to severe burns requiring reconstructive surgery, whereas our study only assessed the prevalence in patients with severe burns (% TBSA burned ≥20). Specifically, mean % TBSA burned in patients undergoing reconstructive surgery in the study of Hop et al. was 20.6 ± 18.7 vs. 39.0 ± 14.6 in our study. Given that severe burns result in more severe scarring, this is the most likely reason that reconstructive surgery was more often required in our study population. When interpreting our reported prevalence of reconstructive surgery, it is important to take into account that we included burn admissions from 2009 till 2019, resulting in a minimal follow-up period of three years. Given the fact that reconstructive surgery can follow up to 10 years post burn, our cohort likely contains patients that require scar revision surgeries in the future.

Similar findings on timing of reconstructive surgery were reported. In the study of Hop et al. [31], approximately 25% of all reconstructions were performed within one year post burn. In our study population, 28.4% of reconstructive admissions were reported within one year post burn.

The initial aim of this study with regard to scarring was to evaluate the short- and long-term quality of scars after burns. As adequate numbers of POSAS scores of patients was only available up to 12 months post burn and the sample only consisted of patients in one burn centre, our results do not give conclusive insights on scar outcomes beyond one year post burn. Nevertheless, we did establish that the cohort of patients with severe burns in the scar registry had similar characteristics as the patient group analysed from the national database with regard to age, sex, TBSA and baux scores. In our study, we found a significant difference in POSAS scores given by observers up to 9–15 months (time point 3) between patients with severe burns versus less severe burns. The non-significant outcome in scores given by patients could be explained by differences in frame of reference for patients and observers, where patients have experience with their specific scar, and observers with many different scars, as noted earlier by Hoogewerf et al. [37].

General improvement of the scar was observed after 3 months, which is consistent with findings in earlier studies [33, 38]. Our findings on reduced scar quality in patients with higher percentages TBSA burned are also reported in studies assessing scar quality [33, 34] As data on reconstructive surgeries performed within the patients in the scar registry was not readily available, the relationship between this indicator and scar severity could not be determined. Preferably, future studies will link the outcome of scar quality to the specific treatment methods used in both the acute and reconstructive phase of care, as this could serve as a long-term outcome measurement of burn treatment. Similarly, linking treatment modality, anatomical location and outcome could provide further valuable insights in burn care.

This study has several other limitations regarding design and the available data that should be mentioned. Patients who were treated but did not survive, were not included in the analysis, as our main goal was to analyse health care utilization and treatment characteristics. Furthermore, we only recorded reconstructive surgery performed in the three dedicated burn centres. Patients seeking reconstructive surgery elsewhere were therefore not included in this analysis, which may have led to underreporting of reconstructive procedures. Furthermore, the follow up of these patients was minimally three years. Thus, later reconstructive procedures may have gone unnoticed for part of the population that was analysed. For the scar registry, we note that the numbers of measurements decline with longer follow up times. This is likely related to patients not returning to the scar clinic if they do not experience complaints from their scars. This might cause more severe cases being overrepresented in the late follow up measurements. Despite the described limitations, our results are consistent with previously conducted studies [22, 28, 33, 34] and provide further insight into a relatively scarce dataset in literature on health care utilization, treatment characteristics and outcomes in severely burned patients.

In conclusion, our data provide a benchmark for health care utilization, treatment characteristics and outcomes for severely burned patients over a prolonged period of time. The impact of our data lies in the detailed analysis of health care utilization and outcome for this specific group of patients with severe burns. Despite the fact that the data may seem straightforward, the availability of such data is very limited in literature. With the growing demand for cost effectiveness data of (novel) health care treatments, this type of baseline data is imperative.

The data indicate specific areas to which improvements in care could be directed, such as the high frequency of reconstructive procedures for contractures in upper extremities. Future innovations in treatment modalities can use these real-world data to demonstrate improvements.

## Supporting information

S1 Fig One minus survival curve. Kaplan-Meier one-minus-survival analyses of time to first reconstructive surgery among survivors. The time to event variable was the interval between the date/year of injury to the date of the event or the last follow-up time (31-12-2022), whichever occurred first.
(TIF)

S1 Table Scar outcome mean POSAS scores. (DOCX)

## References

[1] Buta MR, Donelan MB. Evolution of Burn Care: Past, Present, and Future. Vol. 51, Clinics in Plastic Surgery. W.B. Saunders; 2024. p. 191–204. 38429043 10.1016/j.cps.2023.10.002

[2] Orbay H, Corcos AC, Ziembicki JA, Egro FM. Challenges in the Management of Large Burns. Vol. 51, Clinics in Plastic Surgery. W.B. Saunders; 2024. p. 319–27. 38429052 10.1016/j.cps.2023.11.007

[3] Jeschke MG, Wood FM, Middelkoop E, Bayat A, Teot L, Ogawa R, et al. Scars. Nat Rev Dis Primers. 2023 Dec 1;9(1). doi: 10.1038/s41572-023-00474-x 37973792

[4] Kraft R, Herndon DN, Al-Mousawi AM, Williams FN, Finnerty CC, Jeschke MG. Burn size and survival probability in paediatric patients in modern burn care: A prospective observational cohort study. The Lancet. 2012;379(9820):1013–21. doi: 10.1016/S0140-6736(11)61345-7 22296810 PMC3319312

[5] Spronk I, Polinder S, van Loey NEE, van der Vlies CH, Pijpe A, Haagsma JA, et al. Health related quality of life 5–7 years after minor and severe burn injuries: a multicentre cross-sectional study. Burns. 2019 Sep 1;45(6):1291–9. doi: 10.1016/j.burns.2019.03.017 31174971

[6] Greenhalgh DG. Management of Burns. Longo DL, editor. New England Journal of Medicine [Internet]. 2019 Jun 13;380(24):2349–59. Available from: http://www.nejm.org/doi/10.1056/NEJMra1807442 31189038 10.1056/NEJMra1807442

[7] Oosterwijk AM, Mouton LJ, Schouten H, Disseldorp LM, van der Schans CP, Nieuwenhuis MK. Prevalence of scar contractures after burn: A systematic review. Vol. 43, Burns. Elsevier Ltd; 2017. p. 41–9. 27639820 10.1016/j.burns.2016.08.002

[8] Schouten HJ, Nieuwenhuis MK, van Baar ME, van der Schans CP, Niemeijer AS, van Zuijlen PPM. The degree of joint range of motion limitations after burn injuries during recovery. Burns. 2022 Mar 1;48(2):309–18. doi: 10.1016/j.burns.2021.01.003 34955294

[9] Tapking C, Panayi A, Haug V, Palackic A, Houschyar KS, Claes KEY, et al. Use of the modified meek technique for the coverage of extensive burn wounds. Burns. 2024 May 1; doi: 10.1016/j.burns.2024.01.005 38383170

[10] Rijpma D, Claes K, Hoeksema H, de Decker I, Verbelen J, Monstrey S, et al. The Meek micrograft technique for burns; review on its outcomes: Searching for the superior skin grafting technique. Vol. 48, Burns. Elsevier Ltd; 2022. p. 1287–300. 35718572 10.1016/j.burns.2022.05.011

[11] Asuku M, Yu TC, Yan Q, Böing E, Hahn H, Hovland S, et al. Split-thickness skin graft donor-site morbidity: A systematic literature review. Burns. 2021 Nov 1;47(7):1525–46. doi: 10.1016/j.burns.2021.02.014 33781633

[12] Bache SE, Martin L, Malatzky D, Nessler M, Frank A, Douglas HE, et al. First do no harm: A patient-reported survey of split skin graft donor site morbidities following thin and super-thin graft harvest. Burns. 2024 Feb 1;50(1):41–51. doi: 10.1016/j.burns.2023.10.016 38008702

[13] Lisiecki JL, Buta MR, Taylor S, Tait M, Farina N, Levin J, et al. EFFICACY OF MEPILEX ® AG VERSUS XEROFORM ® AS A SPLIT-THICKNESS SKIN GRAFT DONOR SITE DRESSING: BAD HABITS DIE HARD. Annals of Burns and Fire Disasters. 2023. PMC11041881 38680433

[14] Meuli M, Hartmann-Fritsch F, Hüging M, Marino D, Saglini M, Hynes S, et al. A Cultured Autologous Dermo-epidermal Skin Substitute for Full-Thickness Skin Defects: A Phase I, Open, Prospective Clinical Trial in Children. Plast Reconstr Surg. 2019 Jul 1;144(1):188–98. doi: 10.1097/PRS.0000000000005746 31246829

[15] Germain L, Larouche D, Nedelec B, Perreault I, Duranceau L, Bortoluzzi P, et al. Autologous bilayered self-assembled skin substitutes (Sasss) as permanent grafts: A case series of 14 severely burned patients indicating clinical effectiveness. Eur Cell Mater. 2018 Jul 1;36:128–41. doi: 10.22203/eCM.v036a10 30209799

[16] Shevchenko R V., James SL, James SE. A review of tissue-engineered skin bioconstructs available for skin reconstruction. Vol. 7, Journal of the Royal Society Interface. Royal Society; 2010. p. 229–58. doi: 10.1098/rsif.2009.0403 19864266 PMC2842616

[17] Böttcher-Haberzeth S, Biedermann T, Reichmann E. Tissue engineering of skin. Vol. 36, Burns. 2010. p. 450–60. doi: 10.1016/j.burns.2009.08.016 20022702

[18] Boyce ST, Simpson PS, Rieman MT, Warner PM, Yakuboff KP, Bailey JK, et al. Randomized, Paired-Site Comparison of Autologous Engineered Skin Substitutes and Split-Thickness Skin Graft for Closure of Extensive, Full-Thickness Burns. In: Journal of Burn Care and Research. Lippincott Williams and Wilkins; 2017. p. 61–70. doi: 10.1097/BCR.0000000000000401 27404165 PMC5332328

[19] Golinski P, Menke H, Hofmann M, Valesky E, Butting M, Kippenberger S, et al. Development and characterization of an engraftable tissue-cultured skin autograft: Alternative treatment for severe electrical injuries? Cells Tissues Organs. 2015 Sep 24;200:227–39. 10.1159/000433519 26303436

[20] Kenny EM, Lagziel T, Hultman CS, Egro FM. Skin Substitutes and Autograft Techniques: Temporary and Permanent Coverage Solutions. Vol. 51, Clinics in Plastic Surgery. W.B. Saunders; 2024. p. 241–54. doi: 10.1016/j.cps.2023.12.001 38429047

[21] Cartotto R, Johnson LS, Savetamal A, Greenhalgh D, Kubasiak JC, Pham TN, et al. American Burn Association Clinical Practice Guidelines on Burn Shock Resuscitation. Journal of Burn Care & Research. 2023 Dec 5; 10.1093/jbcr/irad125 38051821

[22] Brusselaers N, Monstrey S, Vogelaers D, Hoste E, Blot S. Severe burn injury in europe: A systematic review of the incidence, etiology, morbidity, and mortality. Crit Care. 2010 Oct 19;14(5). doi: 10.1186/cc9300 20958968 PMC3219295

[23] Dokter J, Vloemans AF, Beerthuizen GIJM, Van Der Vlies CH, Boxma H, Breederveld, et al. Epidemiology and trends in severe burns in the Netherlands. Burns. 2014;40(7). doi: 10.1016/j.burns.2014.03.003 24703338

[24] Smolle C, Cambiaso-Daniel J, Forbes AA, Wurzer P, Hundeshagen G, Branski LK, et al. Recent trends in burn epidemiology worldwide: A systematic review. Vol. 43, Burns. Elsevier Ltd; 2017. p. 249–57. doi: 10.1016/j.burns.2016.08.013 27600982 PMC5616188

[25] Zavlin D, Chegireddy V, Boukovalas S, Nia AM, Branski LK, Friedman JD, et al. Multi-institutional analysis of independent predictors for burn mortality in the United States. Burns Trauma. 2018 Dec 1;6. doi: 10.1186/s41038-018-0127-y 30151396 PMC6103989

[26] Jaspers MEH, Moortgat P. Objective Assessment Tools: Physical Parameters in Scar Assessment. In: Textbook on Scar Management. Springer International Publishing; 2020. p. 149–58. 36351114

[27] Da Costa PTL, Echevarriá-Guanilo ME, Goncąlves N, Girondi JBR, Da Costa Goncąlves A. Subjective tools for burn scar assessment: An integrative review. Vol. 34, Advances in Skin and Wound Care. Lippincott Williams and Wilkins; 2021. p. 1–10. 10.1097/01.ASW.0000749732.09228.a9 33979826

[28] Klein MB, Goverman J, Hayden DL, Fagan SP, McDonald-Smith GP, Alexander AK, et al. Benchmarking outcomes in the critically injured burn patient. Ann Surg. 2014;259(5):833–41. doi: 10.1097/SLA.0000000000000438 24722222 PMC4283803

[29] Kruger E, Kowal S, Pinar Bilir S, Han E, Foster K. Relationship between patient characteristics and number of procedures as well as length of stay for patients surviving severe burn injuries: Analysis of the american burn association national burn repository. Journal of Burn Care and Research. 2020;41(5):1037–44. doi: 10.1093/jbcr/iraa040 32221517 PMC7510847

[30] Carter JE, Amani H, Carter D, Foster KN, Griswold JA, Hickerson WL, et al. Evaluating Real-World National and Regional Trends in Definitive Closure in U.S. Burn Care: A Survey of U.S. Burn Centers. Journal of Burn Care and Research. 2022 Jan 1;43(1):141–8. doi: 10.1093/jbcr/irab151 34329478 PMC8737084

[31] Hop MJ, Langenberg LC, Hiddingh J, Stekelenburg CM, Van Der Wal MBA, Hoogewerf CJ, et al. Reconstructive surgery after burns: A 10-year follow-up study. Burns. 2014;40(8). 10.1016/j.burns.2014.04.014 24927990

[32] van Baar M. Epidemiology of scars and their consequences: burn scars. Téot, Mustoe TA, Middelkoop E, Gauglitz G, editors. Textbook on Scar Management—State of the Art Management and Emerging Technologies. Springer; 2020. p. 37–43. 36351054

[33] Van Der Wal MBA, Vloemans JFPM, Tuinebreijer WE, Van De Ven P, Van Unen E, Van Zuijlen PPM, et al. Outcome after burns: An observational study on burn scar maturation and predictors for severe scarring. Wound Repair and Regeneration. 2012;20(5). 10.1111/j.1524-475X.2012.00820.x 22985039

[34] Wallace HJ, Fear MW, Crowe MM, Martin LJ, Wood FM. Identification of factors predicting scar outcome after burn injury in children: A prospective case-control study. Burns Trauma. 2017 Dec 1;5(1). doi: 10.1186/s41038-017-0084-x 28680887 PMC5494810

[35] Draaijers LJ, Tempelman FRH, Botman YAM, Tuinebreijer WE, Middelkoop E, Kreis RW, et al. The Patient and Observer Scar Assessment Scale: A reliable and feasible tool for scar evaluation. Plast Reconstr Surg. 2004;113(7). doi: 10.1097/01.prs.0000122207.28773.56 15253184

[36] Edgar MC, Bond SM, Jiang SH, Scharf IM, Bejarano G, Vrouwe SQ. The Revised Baux Score as a Predictor of Burn Mortality: A Systematic Review and Meta-Analysis. Journal of Burn Care & Research. 2023 Nov 2;44(6):1278–88. doi: 10.1093/jbcr/irad075 37220881

[37] Hoogewerf CJ, Van Baar ME, Middelkoop E, Van Loey NE. Patient reported facial scar assessment: Directions for the professional. Burns. 2014;40(2). doi: 10.1016/j.burns.2013.07.015 24138808

[38] Goei H, van der Vlies CH, Tuinebreijer WE, van Zuijlen PPM, Middelkoop E, van Baar ME. Predictive validity of short term scar quality on final burn scar outcome using the Patient and Observer Scar Assessment Scale in patients with minor to moderate burn severity. Burns. 2017 Jun 1;43(4):715–23. doi: 10.1016/j.burns.2016.10.012 28040371

