# Supplementary figures and images for: Characteristics, treatments and outcomes in patients with severe burn wounds; a 10 year cohort study on acute and reconstructive treatment
Source: PLoS One. 2024 Nov 22;19(11):e0313287. doi: 10.1371/journal.pone.0313287 (PMC11584074; doi:10.1371/journal.pone.0313287)

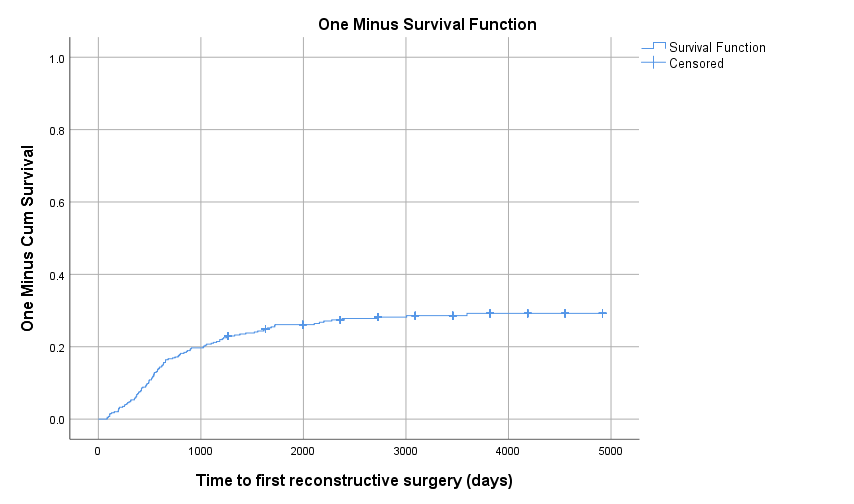

Supplement: S1 Fig — Kaplan-Meier one-minus-survival analyses of time to first reconstructive surgery among survivors. The time to event variable was the interval between the date/year of injury to the date of the event or the last follow-up time (31-12-2022), whichever occurred first. (TIF) [file pone.0313287.s001.tif]
