# Supplementary material for: Characteristics, treatments and outcomes in patients with severe burn wounds; a 10 year cohort study on acute and reconstructive treatment
Source: PLoS One. 2024 Nov 22;19(11):e0313287. doi: 10.1371/journal.pone.0313287 (PMC11584074; doi:10.1371/journal.pone.0313287)
Supplement: S1 Table — (DOCX) [file pone.0313287.s002.docx]

| Supporting Table 1 –Scar outcome mean POSAS scores | | | | | | | | |
| --- | --- | --- | --- | --- | --- | --- | --- | --- |
|  | Total  *n=527* | TBSA 0-20  *n=468* | | TBSA ≥20  *n=59* | | | | P-value |
| **Observer evaluated *(n, %)***  At T1  At T2  At T3  At T4  **Patient evaluated *(n, %)***  At T1  At T2  At T3  At T4 | 360 (68.3)  322 (61.1)  189 (35.9)  47 (8.92)  359 (68.1)  317 (60.2)  184 (34.9)  47 (8.92) | | 321 (68.6)  289 (61.8)  156 (33.3)  37 (7.91)  320 (68.4)  285 (60.9)  152 (32.5)  37 (7.91) | | 39 (66.1)  33 (55.9)  33 (55.9)  10 (16.9)  39 (66.1)  32 (54.2)  32 (54.2)  10 (16.9) | | |  |
| **Mean time post burn ± SD (months)**^a^  At T1 ^a^  At T2  At T3  At T4 | 3.08 ± 0.68  6.10 ± 1.00  11.9 ± 1.47  17.4 ± 1.66 | | 3.10 ± 0.69  6.12 ± 0.99  11.9 ± 1.46  17.4 ± 1.57 | | | 2.99 ± 0.58  5.92 ± 1.14  11.8 ± 1.53 17.6 ± 2.05 | | 0.458  0.191  0.607  0.871 |
| **Mean POSAS score ± SD, observer**  At T1  At T2  At T3  At T4 | 3.12 ± 0.87  3.04 ± 1.00  2.83 ± 1.18  2.74 ± 0.91 | | 3.00 ± 0.80  2.97 ± 0.97  2.68± 1.14  2.65± 0.95 | | | 4.08 ± 0.87  3.69 ± 0.99  3.52 ± 1.16  3.10 ± 0.65 | | 0.000  0.000  0.001  0.062 |
| **Mean POSAS score ± SD, patient**  At T1  At T2  At T3  At T4 | 4.99 ± 1.91  4.51 ± 1.85  3.99 ± 1.87  4.16 ± 1.61 | | 4.97 ± 1.93  4.48 ± 1.88  3.85 ± 1.84  4.05 ± 1.55 | | | 5.16 ± 1.76  4.76 ± 1.59  4.66 ± 1.89  4.53 ± 1.86 | 0.394  0.360  0.020  0.497 | |
| P-values for differences between groups TBSA <20% and TBSA ≥ 20%, calculated with Mann Whitney or ANOVA for categorical data  T1 defined as 0-4.5 month, T2 4.5-9 months, T3 9-15 months, T4 15-21 months  ^a^ Based on 361 values, 324 values, 191 and 48 values for evaluation at T1, T2,T3 and T4 months respectively. | | | | | | | | |
|  | | | | | | | | |
